# Assessing the Histological Malignancy Grade of Olfactory Neuroblastoma Using the Apparent Diffusion Coefficient Histogram Analysis

**DOI:** 10.7759/cureus.66718

**Published:** 2024-08-12

**Authors:** Hideomi Yamauchi, Akira Baba, Ryo Akao, Satoshi Matsushima, Akito Sano, Masaharu Noguchi, Kazuhiro Omura, Teru Ebihara, Nei Fukasawa, Hiroya Ojiri

**Affiliations:** 1 Department of Radiology, The Jikei University School of Medicine, Tokyo, JPN; 2 Department of Otorhinolaryngology, The Jikei University School of Medicine, Tokyo, JPN; 3 Department of Pathology, The Jikei University School of Medicine, Tokyo, JPN

**Keywords:** hyams classification, histogram analysis, adc, dwi, mri, olfactory neuroblastoma

## Abstract

Introduction

Olfactory neuroblastoma (ONB) is a rare malignant tumor of the upper nasal cavity. The Hyams classification is an important histological grading system for diagnosing recurrence and predicting survival in ONB. This study aimed to evaluate the utility of apparent diffusion coefficient (ADC) histogram analysis in distinguishing between high-grade and low-grade ONB based on the Hyams classification system.

Methods

This retrospective study included 17 patients (11 males, six females; mean age 54 years, range 29-84) diagnosed with ONB who underwent pretreatment magnetic resonance imaging (MRI) including diffusion-weighted imaging between December 2017 and September 2022. Two board-certified radiologists outlined the regions of interest on ADC maps of the tumors. Mean, minimum, maximum ADC, standard deviation, skewness, kurtosis, and entropy were calculated from the ADC histograms. Patients were divided into low-grade (Hyams I-II) and high-grade (Hyams III-IV) groups based on histopathological evaluation by a board-certified pathologist. ADC histogram parameters were compared between the two groups using Mann-Whitney U tests. Two-sided p-values of < 0.05 were considered statistically significant.

Results

The study included 10 low-grade (two grade I, eight grade II) and seven high-grade (five grade III, one grade III/IV, one grade IV) ONB cases. Comparison between the low-grade and high-grade groups showed no statistically significant differences in any of the ADC histogram parameters analyzed: mean ADC (median 1.02 vs 0.95; p = 0.591), minimum ADC (0.84 vs 0.78; p = 0.494), maximum ADC (1.06 vs 1.19; p = 0.625), standard deviation (0.09 vs 0.14; p = 0.433), skewness (-0.48 vs -0.75; p = 0.133), kurtosis (2.79 vs 3.12; p = 0.161), and entropy (4.69 vs 5.06; p = 0.315).

Conclusion

This study demonstrated that ADC histogram analysis was unable to differentiate between high-grade and low-grade ONB based on the Hyams classification. The findings suggest that preoperative grading of ONB malignancy using ADC histogram parameters is challenging. Thus, grading based on preoperative imaging evaluation is difficult.

## Introduction

Olfactory neuroblastoma (ONB) is a rare malignant tumor of the upper nasal cavity, accounting for 3% of all sinonasal tumors [1]. The Hyams classification is an important histological grading system for ONB, playing a crucial role in predicting prognosis, guiding the selection of adjuvant therapies, estimating the risk of metastasis, and influencing overall patient management and survival outcomes. This classification categorizes tumors into four groups, ranging from well-differentiated (grade I) to poorly differentiated (grade IV). These categories are generally divided into low grades, consisting of grades I and II, and high grades, consisting of grades III and IV [2,3]. Furthermore, a meta-analysis reported that in ONB, high-grade tumors have more neck and distant metastases and lower survival rates than low-grade tumors [2]. Endoscopy and biopsy are the principal diagnostic steps before treatment, with surgical treatment recommended if ONB is considered resectable after pathological confirmation [4]. However, pathologists may disagree with the pathological diagnosis of malignant grades due to small pretreatment biopsy specimens and variability in results by biopsy site [4]. Therefore, preoperative prediction of malignant status is clinically important for determining treatment strategies and estimating the risk of recurrence in patients with ONB. The apparent diffusion coefficient (ADC) value calculated from diffusion-weighted imaging (DWI), one of the magnetic resonance imaging (MRI) sequences, is known to reflect tumor microstructure, including tumor cellularity and the extracellular matrix [5-7]. The ADC histogram analysis provides information on the distribution of ADC values by plotting the frequency of various ADC values within a region of interest (ROI) and has been reported to be useful in the imaging diagnosis of head and neck tumors [8-11]. To date, no previous study has reported the prediction of the Hyams classification of ONB using ADC histogram analysis. This study aimed to evaluate the value of ADC-based histogram analysis in distinguishing between high- and low-grade ONB.

## Materials and methods

This study received Institutional Review Board approval (The Jikei University School of Medicine, Tokyo, IRB No. 35-172(11801)).

Patients

Patients diagnosed with ONB who underwent pretreatment MRI, including DWI, at our institution between December 2017 and September 2022 were included. We excluded 34 patients who did not have pretreatment MRI performed at our institution. One case of low-volume lesion in which no lesion could be detected on MRI was excluded. Another case was excluded because the possibility of teratocarcinosarcoma could not be ruled out upon pathological reevaluation.

MRI acquisition

MRI was performed using 1.5-T and 3.0-T systems (Symphony, Skyra, Avanto, Siemens Healthineers, Erlangen, Germany) equipped with a 20-channel head-neck coil. All fields of view (FOV) were set in the head and neck regions. The protocol included the following sequences: Echo-planar DWI was obtained using the following parameters-TR/TE, 3,100 ms/82 ms; flip angle, 90°; b value 50, 800 s/mm^2^; field of view, 24 × 24 cm; matrix size, 128 × 128; slice thickness, 7 mm; gap, 3.5 mm; and number of excitations (NEX), 5. ADC maps were automatically generated on the operating console.

ADC histogram analysis

**Figure 1:**
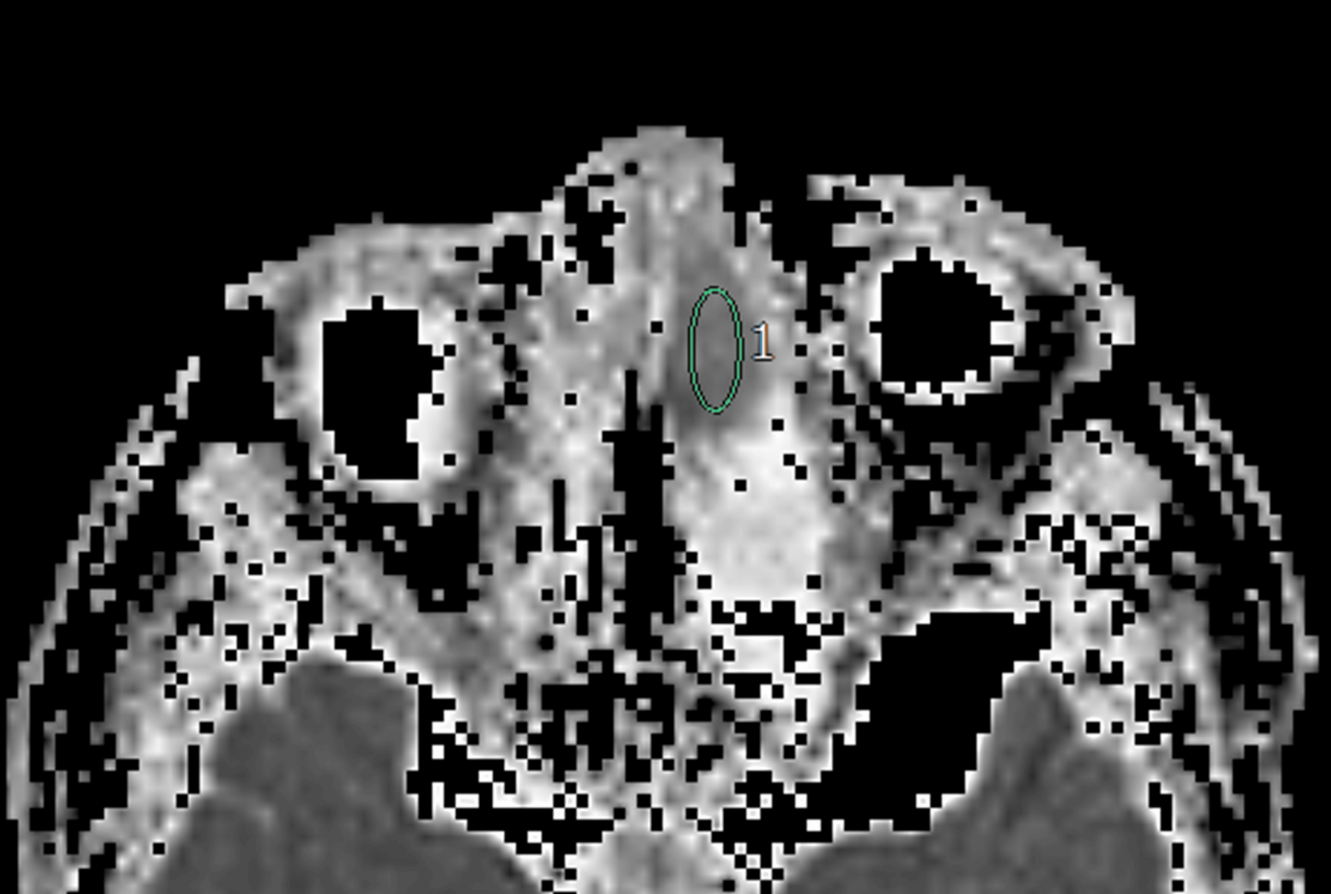
Olfactory neuroblastoma (Hyams III) The apparent diffusion coefficient (ADC) map shows a region of interest in the left nasal cavity lesion. The mean, minimum, maximum, standard deviation, skewness, kurtosis, and entropy of the ADCs were 1.11, 0.99, 1.29, 0.09, -0.753, 3.12, and 4.131, respectively

**Figure 2:**
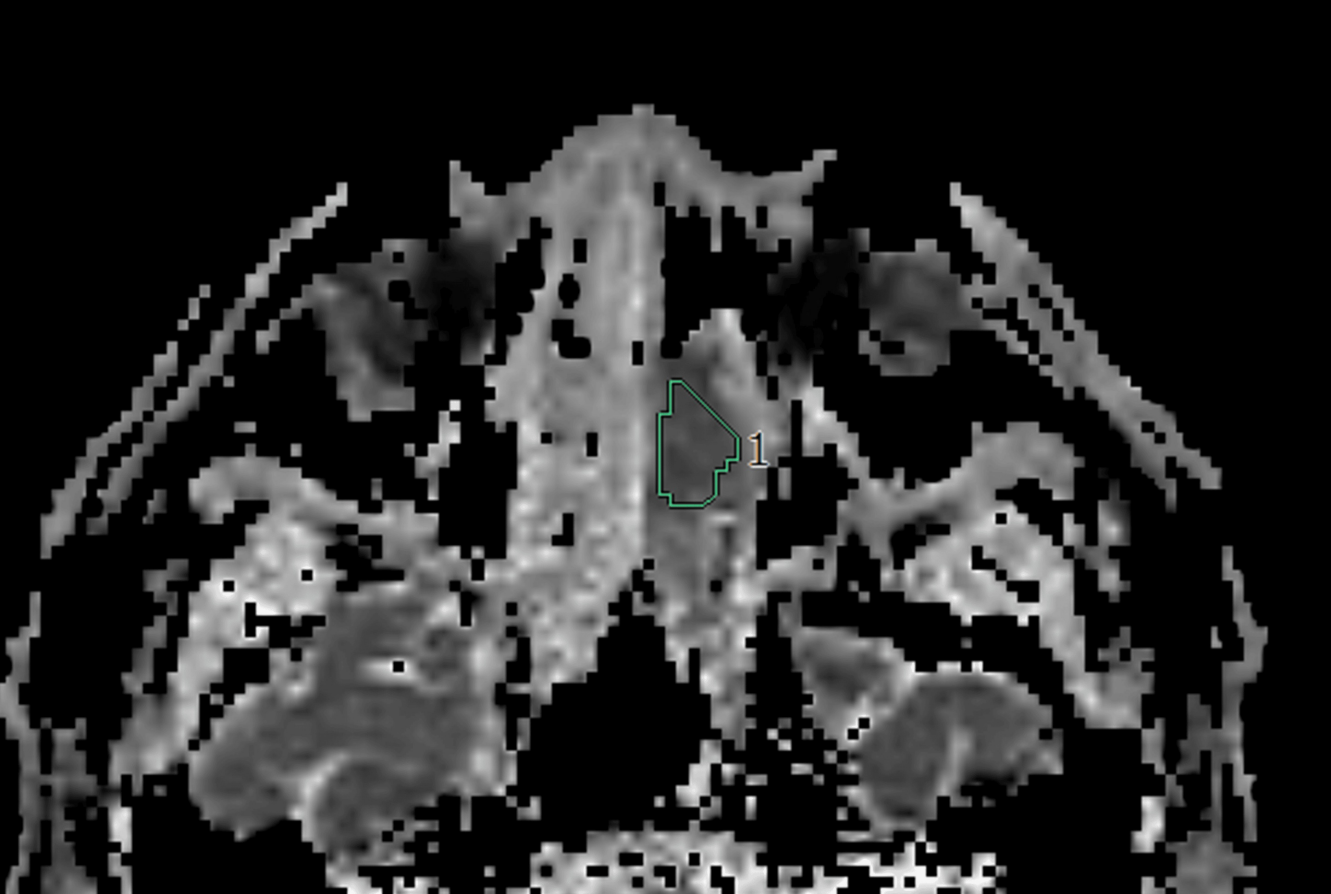
Olfactory neuroblastoma (Hyams II) The apparent diffusion coefficient (ADC) map shows a region of interest in the left nasal cavity lesion. The mean, minimum, maximum, standard deviation, skewness, kurtosis, and entropy of the ADCs were 0.75, 0.61, 0.94, 0.1, -0.54, 2.869, and 4.771, respectively

ADC maps were constructed using an image analysis system (SYNAPSE VINCENT; FUJIFILM Medical Co., Ltd., Tokyo, Japan). Two board-certified radiologists, with seven and 15 years of experience, respectively, outlined two-dimensional ROIs on the ADC maps. The ROIs were drawn on the slice where the lesion was most clearly detectable on the ADC map. The radiologists primarily included the low-signal region while excluding prominent necrotic areas from the ROIs, as determined by consensus (Figures 1-2). For each ROI, the mean, minimum, maximum ADC, standard deviation (SD), skewness, kurtosis, and entropy of ADC were automatically calculated.

Histological grading of ONB

All cases were retrospectively evaluated by a board-certified pathologist with nine years of experience, according to previous reports on pathological resection specimens as follows [2,3,12]: low-grade group ( grades I and II according to Hyams classification) and high-grade group (grades III and IV according to Hyams classification).

Statistical analyses

Shapiro-Wilk tests were performed to confirm that not all data were normally distributed. The Mann-Whitney U test was used to compare ADC histogram parameters between the low- and high-grade ONB groups. The association between Kadish classification and ONB Hyams grading was assessed using Fisher's exact test. Two-sided p-values of < 0.05 were considered statistically significant. All statistical analyses were performed using R version 3.6.1 (R Foundation for Statistical Computing, Vienna, Austria).

## Results

Seventeen patients (11 males and six females) with ONB were included in this study. The mean patient age was 54 years (age range: 29-84 years). Hyams classification was grade I in two cases, II in eight cases, III in five cases, III/IV in one case, and IV in one case. Ten cases were in the low-grade group, and seven were in the high-grade group. Seven cases were classified as A, four as B, and six as C according to the Kadish classification, based on surgical findings and pathological evaluation.

ADC histogram analysis parameters

Table 1 summarizes the results of the ADC histogram analyses. Comparison between the low- and high-grade groups of ONB showed no significant differences in the following ADC parameters: mean ADC (median 1.02 vs. 0.95; p = 0.591), minimum ADC (median 0.84 vs. 0.78; p = 0.494), maximum ADC (median 1.06 vs. 1.19; p = 0.625), SD of ADC (median 0.09 vs. 0.14; p = 0.433), skewness of ADC (median -0.48 vs. -0.75; p = 0.133), kurtosis of ADC (median 2.79 vs. 3.12; p = 0. 161), and the entropy of ADC (median 4.69 vs. 5.06; p = 0.315). The distribution of high-grade to low-grade ONB did not differ significantly across Kadish classifications (A: 2/5, B: 3/1, C: 2/4; p = 0.33).

**Table 1:** ADC histogram parameters of olfactory neuroblastoma ADC: Apparent diffusion coefficient. Data are median and range. ^a^Mann-Whitney U test test was used (significance considered at p < 0.05)

ADC parameters	Low-grade olfactory neuroblastoma	High-grade olfactory neuroblastoma	p-value^a^
Mean	1.02 (0.75-1.11)	0.95 (0.63-1.11)	0.59
Minimum	0.84 (0.61-0.91)	0.78 (0.39-0.99)	0.49
Maximum	1.06 (0.93-1.48)	1.19 (1.03-1.30)	0.63
Standard deviation	0.09 (0.06-0.22)	0.14 (0.09-0.19)	0.43
Skewness	-0.48 (-1.06--0.48)	-0.75 (-3.22--0.43)	0.133
Kurtosis	2.79 (1.95-4.54)	3.12 (2.83-20.4)	0.16
Entropy	4.69 (3.42-5.40)	5.06 (3.55-5.66)	0.32

## Discussion

In this study, we compared the parameters obtained from the pretreatment ADC histogram analysis of ONB between high- and low-grade groups, and no significant differences were observed between the two ONB groups.

ONB is a malignant neuroectodermal tumor arising in the nasal cavity's olfactory epithelium and was first described by Berger et al. in 1924. ONB is a rare disease, accounting for approximately 3% of all nasal sinus tumors, and is slightly more common in males. In the present study, similar to previous reports, the incidence of ONB was higher in males. ONB has been reported to have a bimodal age distribution [13]. However, more recent large-scale studies have demonstrated a true unimodal distribution, with a peak between the fourth and sixth decades [14]. In the present study, no bimodal distribution was observed, and as in previous reports, the distribution was unimodal, with a peak in the fifth decade.

The Hyams classification is often used for histopathological evaluation of ONB. The Hyams grading system based on histological maturation and differentiation has been shown to be of prognostic value, particularly in complementing current staging systems [12,15,16]. Grades I and II highly differentiated tissues have foci-like structures surrounded by neurofibrils around the nucleus. Conversely, poorly differentiated Grades III and IV tissues show disorganized foci-like structures and many necrotic cells. Multiple studies have shown that the Hyams grade allows the identification of aggressive locoregional disease and subsequent prediction of poor disease-free survival and may enable stratification for adjuvant therapy [15,17-19]. Furthermore, a recent meta-analysis reported that compared to low-grade Hyams ONB, high-grade Hyams ONB was associated with higher rates of both neck and distant metastases, as well as overall survival [2].

DWI sequences with ADC maps are currently part of the standard head and neck cancer study protocols. ADC maps can be easily segmented and expressed as mean values or, in more detail, as described by first-order statistics (histogram analysis). Histogram analysis of the ADC map revealed the following parameters: mean, SD, kurtosis, skewness, and entropy [20]. “Kurtosis” indicates the histogram peakedness (the lower the kurtosis, the more flattened the histogram); “skewness” is related to histogram symmetry (positive skewness indicates a right-tailed histogram); and “entropy” is a metric positively associated with image heterogeneity. The usefulness of ADC histograms in the head and neck region has been reported for monitoring early treatment-induced changes in head and neck squamous cell carcinoma [7], estimating histopathological parameters related to prognosis in head and neck squamous cell carcinoma [9,10], and estimating histological types of parotid gland tumors [11].

Based on the above, it was hypothesized that ADC histograms might be able to assess the microscopic features of tumors. However, in this study, it was difficult to distinguish high-grade ONB from low-grade ONB. Previous studies have reported no significant differences in histogram analysis or multiparameter texture analysis, including ADC comparisons, in differentiating parotid cancer grades, and it has been argued that parotid cancer grade is difficult to assess using pretreatment image characteristics [21]. Similar to the findings of that study, it was difficult to estimate the malignant potential of ONB through pretreatment image evaluation in this study. In ADC histogram analysis, pretreatment grading of ONB is difficult and is evaluated by surgical resection of the pathology; therefore, evaluating lesion progression and spread is more important as a diagnostic imaging tool for adequate treatment.

This study has several limitations. This preliminary retrospective study was conducted at a single center with a limited number of patients because of the rare incidence of ONB and the small number of MRIs performed at our institution. The use of 1.5-T 3.0-T MRI systems may have caused heterogeneity in the results.

## Conclusions

This study demonstrated that ADC histogram analysis could not significantly differentiate between high-grade and low-grade ONB based on the Hyams classification. Our findings suggest that preoperative grading of ONB using ADC histogram parameters is challenging. Although ADC analysis remains valuable for evaluating many head and neck tumors, its application in predicting ONB grade appears limited. These results underscore the continued importance of histopathological evaluation in determining ONB grade and guiding treatment decisions. Future research incorporating larger sample sizes and advanced imaging techniques may provide further insights into noninvasive methods for assessing ONB grade.
